# Practical Hints upon Medical Examination for Life Assurance—I

**Published:** 1908-08-01

**Authors:** William P. S. Branson

**Affiliations:** Junior Medical Officer, Sun Life Office.


### Life Assurance.

### PRACTICAL HINTS UPON MEDICAL EXAMINATION FOR LIFE ASSURANCE—I.

By WILLIAM P. S. BRANSON, M.D., M.R.C.P., Junior Medical Officer, Sun Life Office.

Most life assurance offices employ at their chief offices doctors whose business it is to consider the reports received from medical referees throughout the country, and to make final recommendations, based upon these reports, to the managers of the offices they serve. These articles are designed to assist medical referees in the task of making their reports as illuminating as possible to the chief office examiners, and thus as helpful and protective as possible to the societies concerned and to all who hold policies in them. They do not pretend to completeness, but are concerned with common points.

Although from the purely medical point of view no examination of a candidate can be too thorough, in practice the thoroughness of the examination is limited by the need for not making it forbidding. The estimation of the premiums to be charged is based upon averages, and therefore a large number of assured lives is a great element of strength to an office, for the greater the number of lives at risk, the more nearly fixed and calculable is the mortality. A large business is therefore a great desideratum, and the object of all progressive offices is to make life assurance as easy for the public as is consistent with their own security. The routine examination for this reason should not be conducted with too much *finesse*, when this involves discomfort to the proposer, lest people be deterred from assuring their lives by a fear of the ordeal which awaits them at the doctor's hands. The object of the examination is fulfilled by the elimination of the more or less grossly unfit, and particularly of those who, doubting of their health, are anxious to make provision for their dependents. Such selection against an office must be precluded by the doctor's care; but, for the rest, the relatively poor and the relatively good cases are sufficiently balanced, unless the business be numerically small, and the contingencies to be expected come within the scope of the actuarial estimates upon which premiums are calculated. But when the sum to be assured is unusually large, more discrimination is needed; for the larger the sum, the fewer the policies within the category to which it belongs and the greater the moment of every lapse from calculated contingencies. As a general rule, therefore, the examination should be intelligent and watchful, but not too minute; for if there be good ground for the suspecting of an obscure malady likely to endanger life, the case in point ceases to be one with which any society would care to meddle until all doubt has been dissipated. The correct recommendation in all such instances is one of postponement, with a statement of the reasons prompting it.

Prior to the examination the medical referee should read the proposal with attention, for the answers to questions are often so vague or popular as to be useless in themselves. It is a part of his duty to define them and make them as precise as he can. A few queries by word of mouth will often alter completely the complexion of a statement upon the proposal, and obviate much delay and correspondence. A few examples will emphasise the usefulness of such precautions.

A man will state that his occupation is that of a door-keeper. Now the door-keeper of a drapery establishment is a life of ordinary risk; but the door-keeper of a ginshop follows a calling which, from the associated temptations to alcoholic excess, is very properly considered a dangerous one.

The present tendency in the life-assurance world is towards discounting the importance of family histories of disease, especially when the life proposed has reached middle age or passed it. There are, however, three diseases the occurrence of which in the immediate relatives of the life proposed are held to be a factor of importance in estimating the risk. These are the following: (1) Tuberculosis in parents, brothers, or sisters, when the life proposed is below middle age or thereabouts; (2) serious pyschoses affecting more than one immediate relative, including in the category suicide, proved or reasonably presumed; (3) early deaths from degenerative diseases of more than one immediate relative, such, let us say, as apoplexy or angina pectoris in one parent, and Bright's disease in the other, both ending fatally before the age of fifty years.

Family histories, as given in proposals, are notorious for vagueness, the latter arising mostly from ignorance, but not seldom from design. In these statements tuberculosis of the lungs masquerades in many disguises, of which the following are the commonest. Chill, child-birth, influenza, asthma, exposure, bronchitis, pneumonia, broken blood-vessel. In the face of such reported causes of death, given without comment, the chief office adviser is left incompetent to form an opinion. It is therefore incumbent upon the referee, provided that the age of the proposer warrants the investigation, to make detailed inquiry into the nature and duration of the fatal illness. In the case of the two other diseases we have mentioned misstatements are not so frequent, though suicide is fairly commonly attributed to accident.

The record of past illnesses is a constant source of replies whose lack of precision calls for inquiry and comment upon the part of the examiner. For example: The answer "pleurisy" when seen on paper, especially when the life concerned is below middle age, always raises the spectre of tuberculosis; but the exercise of care in definition may well enable the examiner to reach an assured conclusion as to the real nature of the ailment, which may or may not turn out to be of importance. "Influenza," again, is a common synonym for pulmonary tuberculosis, whether admitted or only shrewdly suspected. "Nervous breakdown" quite often does duty for melancholia or delusional insanity; "inflammation" and "colic" for appendicitis, or for symptoms of renal or biliary lithiasis. From time to time the misuse of a technical term, such as "neuritis" for "nephritis," is a potential source of false judgments upon the value of a symptom like slight albuminuria. It is not necessary to multiply examples in this matter. The reader will not fail to appreciate the need for threshing out the truth from the dubious records of past affections.

The two diseases most dreaded by life officers are pulmonary tuberculosis and alcoholism, for these are responsible for a large proportion of early deaths. It is thus of the highest moment that addiction to alcohol should not pass undetected, especially in the case of male lives after middle age. Direct inquiry as to the average daily consumption of alcohol, though not by any means to be neglected, is not always sufficient in itself, for the answer is least likely to be truthful when truth is most essential. For this reason the answer given must be read in the light of the proposer's appearance, his manner, his dress, and the existence or absence of corroborative evidence of alcoholism such as tremor. It may be remarked in passing that as a general rule alcoholic excess leaves its mark upon men with far greater prominence than is the case with women. This at least is so among the life-assuring classes. It may be correlated with the fact that such women confine themselves to spirits, while men of the same class are more catholic in their tastes. However it be, there is no doubt that the detection of tippling habits among women is a matter of great difficulty.

*(To be continued.)*

